# COVID-19 in real world: Survival and medical costs of hospitalized patients in Brazil´s first wave

**DOI:** 10.1016/j.bjid.2023.102778

**Published:** 2023-05-15

**Authors:** Jaime Luís Lopes Rocha, Irina Riediger, Juliano Gasparetto, Felipe Francisco Tuon

**Affiliations:** a Pontifícia Universidade Católica do Paraná, Faculdade de Medicina, Laboratório de Doenças Infecciosas Emergentes, Curitiba, PR, Brazil; b Laboratório Central do Estado do Paraná, São José dos Pinhais, PR, Brazil

**Keywords:** COVID-19, Brazil, Survival, Cost, Microcosting, Burden of disease, Economic burden

## Abstract

**Objective:**

To evaluate survival and direct medical costs of patients admitted in private hospitals with COVID-19 during the first wave.

**Methods:**

A retrospective, observational study analyzing survival and the economic data retrieved on hospitalized patients with COVID-19. Data from March 2020 to December 2020. The direct cost of hospitalization was estimated using the microcosting method with each individual hospitalization.

**Results:**

342 cases were evaluated. Median age of 61.0 (95% CI 57.0‒65.0). 194 (56.7%) were men. The mortality rate was higher in the female sex (*p* = 0.0037), ICU (*p* < 0.001), mechanical ventilation (*p*<0.001) and elderly groups. 143 (41.8%) patients were admitted to the ICU (95% CI 36.6%–47.1%), of which 60 (41.9%) required MV (95% CI 34.0%–50.0%). Global LOS presented median of 6.7 days (95% CI 6.0–7.2). Mean costs were US$ 7,060,00 (95% CI 5,300.94–8,819,00) for each patient. Mean cost for patients discharged alive and patients deceased was US$ 5,475.53 (95% CI 3,692.91–7,258.14) and US$ 12,955.19 (95% CI 8,106.61–17,803.76), respectively (*p* < 0.001).

**Conclusions:**

Patients admitted with COVID-19 in these private hospitals point to great economic impact, mainly in the elderly and high-risk patients. It is key to better understand such costs in order to be prepared to make wise decisions during the current and future global health emergencies.

## Introduction

The novel coronavirus, SARS-CoV-2, is the causative agent of the infectious disease commonly named COVID-19. It was first described in December 2019 in Wuhan, China and it was first detected in Brazil in March 2020.^1^^,^^2^

According to WHO data, there were at least 82.6 million COVID-19 cases reported by the end of December 2020, with 1.8 million deaths.^3^ During the same period, Brazil had 7.6 million cases and almost 200 thousand deaths.^4^ By the end of July 2021, there have been near 200 million cases with 4.2 million deaths worldwide. Meanwhile, Brazil accounted for almost 20 million cases and 558 thousand deaths by the end of July.^3^^,^^4^

From an economic point of view, COVID-19 has imposed high direct and indirect costs on all perspectives.^5^, ^6^, ^7^, ^8^ For all medical conditions, direct medical costs are affected predominantly by the number of infected people, but also by mean length of stay in hospital, mainly in Intensive Care Unit (ICU), and mechanical ventilation.^9^^,^^10^ However, it seems that medical costs are higher for COVID-19 patients than costs for other infectious diseases, which may be partially explained by the number of COVID-19 patients who may need hospital care assistance, which is around 3%, and the fact that 50% to 88% of these hospitalized patients may need invasive ventilation.^5^^,^^9^^,^^11^^,^^12^

Indirect costs will be colossal and previous studies had already predicted only for 2020 an average economic impact of −4.5% of Gross Domestic Product (GDP), because there are many channels through which COVID-19 pandemic influences the economy.^13^ Moreover, traditional health economics metrics may underestimate the true costs of the current crisis, which have shown to be worse than previous epidemics.^13^ For example, a web-based survey conducted in the beginning of the pandemic with more than 45,000 Brazilian volunteers reported that 55% presented a decrease in family income, with the group of informal workers being the most affected.^14^ Moreover, according to the official records, from February 2020 to January 2021 there were 98,025 deaths from COVID-19 in Brazil which account for 1.2 million Years of Potential Life Lost (YPLL).^15^ The burden of disease was different according to gender, race, age but it was mostly affected by socioeconomic vulnerability, at least in the beginning of pandemic.^16^^,^^17^

Cost of illness studies have been used to draw public attention to a specific condition as well as to help in the evaluation of policy options.^10^ Direct costs reflect all resources used for diagnostic and treatment purposes. However, productivity losses and mortality are part of the intangible costs, which are not always immediately available or validated.^10^^,^^18^

Health care in Brazil is a very complex socioeconomic system, which is composed of private and public market elements supposed to respond to private and social interests. The sector represents almost 10% of the country's GDP, of which, the majority is privately provided (56%) and the remaining 44% is public sourced. Private sector provides medical assistance to almost 50 million beneficiaries, but the vast majority (75.6%) of the population relies only on the public sector. While the private sector is financed by public and private resources, mostly for profit, and comprises different modalities of insurance and private health plans, public system suffers from underfunding.^19^

There are no published data on the direct medical costs associated with hospitalization due to COVID-19 in Brazil´s private health services, and there is very little data on public services, despite their importance in economic analysis and policy decision-making. The main objective of this study was to identify the direct medical costs associated with hospitalization due to COVID-19 in Brazil´s private health services.

## Methods

This study is a retrospective, descriptive analysis assessing the economic burden of hospitalized patients with COVID-19 in Curitiba, the capital of Paraná State, southern Brazil. We propose a traditional approach to determine the direct costs of Treatment during hospital admission, which comprises the healthcare-related resources used in patient management from the perspective of the largest private healthcare provider in the country.

COVID-19 cases, confirmed by RT-qPCR, admitted to four major private hospitals in the city, between March 1st and December 31st, 2020, whose costs were attributed to a single private health care provider were enrolled in the study. Patients aged <18 years were excluded from the study. The costs before admission and after discharge were not included in the analysis.

The direct cost of hospitalization was estimated using the microcosting method. Both healthcare claims data and clinical records were used to improve the final analysis, which included all direct costs associated with each individual hospitalization. Readmissions were considered new cases for economic analysis. Total cost was divided into five categories: hospital daily costs (costs of ICU and/or clinical ward bed services, which include nursing services), medical costs (medical procedures and medical visit costs), oxygen costs, medications, and materials (all medications, therapeutic diets, Personal Protective Equipment (PPE), Extracorporeal Membrane Oxygenation (ECMO), and hemodialysis materials), and other costs (laboratory\radiology exams, and blood products). The demographic and clinical data analyzed were age, sex, comorbidities, use of tobacco, Hospital-Acquired complications Sepsis (HAS), Pneumonia (HAP), Pressure Ulcers (PU), falls, pneumothorax, Urinary Tract Infection (UTI), *Clostridium difficile* infection, and non-infectious complications of vascular access), procedures (transfusions, tracheostomy, Hemodialysis (HD), and pleural effusion drainage), Length of Hospital Stay (LOS) including admission to the General Medical Ward (GMW) or to the Intensive Care Unit (ICU), demand for Mechanical Ventilation (MV), and final clinical outcome (discharge or death).

National directives for economic studies in the country were observed and to access sample representativeness, total numbers of hospitalized COVID-19 cases in the same period and geographic area were retrieved from the Brazilian Ministry of Health database.^20^, ^21^, ^22^ Financial data were collected in Brazilian currency (Reais, R$) and converted to US dollars (US$).^23^ During the study period, US$ 1 was worth an average of R$ 5.33.23 The study protocol was approved by the Ethics Review Board Committee (CAAE 57,226,822.6.0000.0020).

Categorical variables were expressed as numbers (n) of cases and proportions (%). Continuous variables were expressed as median (95% Confidence Interval and interquartile range) or mean (standard deviation) and were compared among groups using Kruskal-Wallis tests for independent samples. Four age clusters were predicted for statistical analysis. The LOS of each patient subgroup was used to assess the impact of different variables on hospital costs. The average cost (total cost/number of admissions) and the cost per day (total cost/total follow-up in days) were estimated. The applied hypothesis tests had an alpha error of 0.05. A multiple logistic regression model was proposed to assess the adjusted impact of clinical conditions, procedures, and complications on mortality, and the accuracy of the model was presented as the area under the ROC curve.

Kaplan-Meier (KM) survival analyses were performed to examine the overall survival probability. Moreover, the survival probability was estimated across Mechanical Ventilation (MV) use and sex. Log-rank analysis was performed and considered significant if *p* < 0.05.

Costs will be presented as median (95% CI), but in discussion, the mean (95% CI) may be used to make comparisons with international literature possible.

Statistical analyses were performed using IBM SPSS Statistics (version 26.0; SPSS Inc., Chicago, Illinois, USA).

## Results

Curitiba had 11,256 patients hospitalized due to COVID-19 between March 1st and December 31st, 2020, and 11,090 (98.5%) were aged 18 years or older. Of those, 9.2% (*n* = 1.020) were admitted to one of the four hospitals monitored in this study. There were 342 admissions from a single private healthcare provider that were included in the analysis during the study period, and all these cases were included for survival and economic analysis. There were only five cases of readmission in the sample. The study population had a median age of 61.0 (95% CI 57.0‒65.0) and the interquartile range was 45.0 to 76.0. Of 342 patients, 194 (56.7%) were men. Average age was significantly different for males and females (64.2 ± 20.1 vs. 58.2 ± 17.2; *p* = 0.003).

There were 70 fatalities in the sample (20.5%, 95% CI 16.2–24.8%). The mortality rate was higher in the female sex (*p* = 0.0037), ICU (*p* < 0.001) and MV (*p* < 0.001) groups. The elderly population had higher mortality rates (Table 1).

**Table 1:** Descriptive statistics, including absolute and relative frequencies, according to demographics, clinical conditions, and final outcome.

		Final outcome			
		Discharged	Death	Total	
		n (n%) or Median (95% Cl)	n (n%) or Median (95% Cl)	n (n%) or Median (95% Cl)	
**Gender**	Female	110 (40.4%)	38 (54.2%)	148 (43.2%)	*p* = 0.037
	Male	162 (59.5%)	32 (45.7%)	194 (56.7%)	
**Age Group**	Age in years	55.0 (52.0‒58.0)	83.0 (80.0‒86)	61.0 (57.0‒65.0)	*p* < 0.001
	18 to 40	57 (20.9%)	0	57 (16.6%)	*p* < 0.001
	41 to 60	105 (38.6%)	5 (7.1%)	110 (32.1%)	
	61 to 80	91 (33.4%)	24 (34.2%)	115 (33.6%)	
	Above 80	19 (6.9%)	41 (58.5%)	60 (17.5%)	
	LOS in days	6.1 (5.6‒6.8)	8.8 (7.7‒11.7)	6.7 (6.0‒7.2)	*p* < 0.001
	ICU	96 (67.2%)	47 (32.9%)	143 (100%)	*p* < 0.001
	LOS in ICU in days^a^	3.0 (2.8‒49)	8.5 (5.9‒13.5)	4.7 (3.4‒6.4)	*p* < 0.001
	MV	21 (35.0%)	39 (65.0%)	60 (100%)	*p* < 0.001
	LOS in MV in days^b^	9.1 (5.5‒169)	8.8 (5.9‒12.2)	8.9 (7.0‒12.2)	*p* < 0.001
**Comorbidities**	HBP	109 (40.0%)	40 (57.1%)	149 (43.5%)	*p* = 0.010
	DM	70 (25.7%)	13 (18.5%)	83 (24.2%)	*p* = 0.213
	Cardiopathy	46 (16.9%)	23 (32.8%)	69 (20.1%)	*p* = 0.003
	NeuroPsiq	40 (14.7%)	13 (18.5%)	53 (15.5%)	*p* = 0.426
	Dementia	12 (4.4%)	30 (42.6%)	42 (12.8%)	*p* < 0.001
	Dyslipidemia	34 (12.5%)	8 (11.4%)	42 (12.8%)	*p* = 0.807
	COPD	19 (6.9%)	18 (25.7%)	37 (10.8%)	*p* < 0.001
	Obesity	25 (9.1%)	8 (11.4%)	33 (9.6%)	*p* = 0.572
	Hypothyroidism	21 (7.7%)	4 (5.7%)	25 (7.3%)	*p* = 0.213
	ARF	10 (3.6%)	13 (18.5%)	23 (6.7%)	*p* < 0.001
	Neoplasia	12 (4.4%)	9 (12.8%)	21 (6.1%)	*p* = 0.009
	CRF	12 (4.4%)	5 (7.1%)	17 (4.9%)	*p* = 0.349
	CHF	6 (2.2%)	10 (14.2%)	16 (4.6%)	*p* < 0.001
	Smoking	13 (4.7%)	2 (2.8%)	15 (4.3%)	*p* = 0.484
	Immunodeficiency^c^	6 (2.2%)	0	6 (1.7%)	*p* = 0.210
**Total number of comorbidities**	None	99 (36.3%)	2 (2.8%)	101 (29.5%)	*p* < 0.001
	1	51 (18.7%)	10 (14.2%)	61 (17.8%)	
	2	54 (19.8%)	19 (27.1%)	73 (21.3%)	
	3 or more	68 (25.0%)	39 (55.7%)	107 (31.2%)	
**Complications**	Phebitis	22 (8.0%)	11 (15.7%)	33 (9.6%)	*p* = 0.054
	Pressure Ulcer	7 (2.5%)	18 (25.7%)	25 (7.3%)	*p* < 0.001
	Sepsis	11 (4.4%)	13 (18.5%)	24 (7.0%)	*p* < 0.001
	HAP	3 (1.1%)	8 (11.4%)	11 (3.2%)	*p* < 0.001
	UTI	2 (0.7%)	1 (1.4%)	3 (0.8%)	*p* = 0.579
	Fall	2 (0.7%)	0	2 (0.5%)	*p* = 0.472
	Pneumothorax	1 (0.3%)	1 (1.4%)	2 (0.5%)	*p* = 0.299
	C.diff. Infection	2 (0.7%)	0	2 (0.5%)	*p* = 0.472
**Total number of Complications**	None	238 (87.5%)	42 (60.0%)	280 (81.8%)	*p* < 0.001
	1	24 (8.8%)	14 (20.0%)	38 (11.1%)	
	2 or more	10 (3.6%)	20 (28.5%)	24 (7.0%)	
**Procedures**	Hemodyalisis	6 (2.2%)	14 (20.0%)	20 (5.8%)	*p* < 0.001
	Tracheostomy	6 (2.2%)	7 (10.0%)	13 (3.8%)	*p* = 0.002
	Transfusion	3 (1.1%)	6 (8.5%)	9 (2.6%)	*p* < 0.001
	Pleural drainage	1 (0.3%)	4 (5.7%)	5 (1.4%)	*p* < 0.001
**Total number of procedures**	None	258 (94.8%)	47 (67.1%)	305 (89.1%)	*p* < 0.001
	1	12 (4.4%)	17 (24.2%)	29 (8.4%)	
	2 or more	2 (0.7%)	6 (8.5%)	8 (2.3%)	
**Total**		272 (79.5%)	70 (20.4%)	342 (100%)	

a For patients admitted to ICU (not all patients).

b For patients in MV (not all patients) Smoking (actual or past).

c Including HIV, use of immunosuppressors; p-value based on Mann-Whitney *U* on Kruskal Wallis test for independent samples. 95% CI, 95% Confidence Interval; ICU, Intensive Care Unit; MV, Mechanical Ventilation; LOS, Length of Stay; CHF, Cardiac Heart Failure; HBP, High Blood Pressure; DM, Diabetes Mellitus; COPD, Chronic Obstructive Pulmonary Disease; ARF, Acute Renal Failure; CRF, Chronic Renal Failure; HAP, Hospital Acquired Pneumonia; C. Diff, Clostridium Difficile; UTI, Urinary Tract Infection.

A total of 143 (41.8%) patients were admitted to the ICU (95% CI 36.6–47.1%), of which 60 (41.9%) required MV (95% CI 34.0–50.0%). global LOS presented median of 6.7 days (95% CI 6.0–7.2) and the interquartile range was 3.8 to 10.9 days. The median LOS of the patients admitted to the ICU and submitted to MV was 4.7 and 8.9 days, respectively. Comorbidities, complications, and procedures are presented in Table 1.

**Fig. 1:**
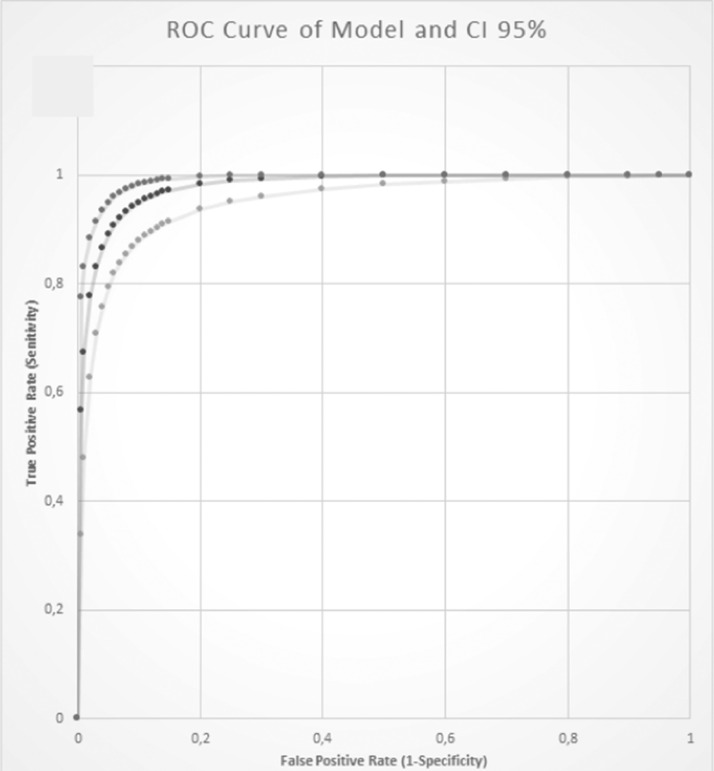
ROC Curve and 95% CI. Area under ROC curve 0.980 (95% CI 0.967–0.992); SE = 0.006; *p* < 0.001.

The multiple logistic regression model is presented in Table 2, and the accuracy is presented as the area under the ROC curve in Fig. 1.

**Table 2:** Multiple logistic regression model to assess the adjusted impact of clinical conditions on mortality.

					Exp(B) 95% CI	
	B	SE	p	Exp(B)	Inferior	Superior
**Age**	0.151	0.034	0.000	1.162	1.087	1.243
**LOS**	−0.264	0.065	0.000	0.768	0.677	0.872
**MV**	4.458	1.032	0.000	86.297	11.428	651.685
**LOS in ICU**	0.197	0.076	0.010	1.217	1.049	1.413
**Dementia**	2.817	0.744	0.000	16.730	3.891	71.933
**COPD**	2.936	0.846	0.001	18.831	3.590	98.785
**Pressure Ulcer**	2.855	1.206	0.018	17.377	1.636	184.558
**Transfusion**	3.397	1.388	0.014	29.889	1.967	454.222
**Hemodyalisis**	3.594	1.115	0.001	36.370	4.089	323.522
**Constant**	−13.607	2.740	0.000	0.000		

Dependent variable: death. Variables included in the model were as follows: Age, LOS (Length of Stay); MV (Mechanical Ventilation), LOS in ICU (Length of Stay in ICU), Dementia, COPD (Chronic Obstructive Pulmonary Disease), Pressure ulcer, Transfusion and Hemodyalisis. B, beta; SE, standard error.

The daily amount of money spent for every single patient was US$ 728.46, but there are major differences according to patient accommodation and the necessity of MV. Median cost, according to demographics, clinical conditions, complications, and procedures during hospital stay and final outcome are presented in Tables 3 and 4. Mean (95% CI) costs were US$ 7060,00 (5300.94–8819.00) for each patient.

**Table 3:** Descriptive statistics with median cost, according to demographics, clinical conditions, and final outcome.

		COSTS in USS			
		Discharged	Death	Total	
		Median (95% Cl)	Median (95% Cl)	Median (95% Cl)	
**Gender**^b^	Female	2088.46 (1692.25‒2849.99)	5773.58 (3855.02‒9363.24)	2849.99 (2155.15‒3714.52)	*p*<0.001
	Male	1473.56 (1301.87‒1885.83)	4845.16 (2940.21‒15,988.14)	1816.34 (1414.38‒2232.29)	*p*<0.001
**Age Group**^a^	18 to 40	1377.33 (935.55‒2020.60)	‒	1377.33 (935.55‒2020.60)	‒
	41 to 60	1749.44 (1345.85‒1927.82)	15,955.13 (15,592.95‒53,724.14)	1816.34 (1432.69‒2021.77)	*p*<0.001
	61 to 80	1808.83 (1402.69‒3122.61)	7051.29 (4618.88‒23,488.41)	2722.95 (1707.87‒3848.95)	*p*<0.001
	Above 80	4498.82 (1976.67‒7476.60)	3813.76 (2832.86‒6009.13)	3861.82 (2849.99‒5799.23)	*p* = 0.946
**ICU**^a^	Non-admitted	1245.52 (1034.40‒1377.33)	2368.55 (1781.72‒3060.10)	1321.52 (1148.21‒1432.69)	*p*<0.001
	Admitted	4838.18 (3654.89‒6217.29)	11,589.96 (6389.92‒15,988.14)	6118.69 (4958.47‒7421.93)	*p* = 0.001
**MV**^a^	Non-submitted	1661.62 (1402.69‒1846.98)	2607.23 (1969.45‒3714.52)	1752.47 (1473.56‒1907.94)	*p* = 0.006
	Submitted	23,776.69 (11,294.61‒50,553.79)	13,887.08 (9524.22‒21,676.50)	15,790.54 (11,589.97‒22,485.54)	*p* = 0.076
**Total number of Comorbidities**^a^	None	1807.94 (1432.69‒2021.77)	18,713.77 (4845.17‒32,583.38)	1824.75 (1433.70‒2050.97)	*p* = 0.032
	1	1385.99 (1021.40‒1705.56)	4179.68 (1569.00‒9363.24)	1533.23 (1211.99‒1885.83)	*p* = 0.009
	2	1426.53 (1148‒2569.12)	7385.97 (3815.56‒23,596.21)	2480.06 (1429.77‒3738.47)	*p*<0.001
	3 or more	3256.44 (1744.71‒5002.24)	4788.67 (2940.21‒7421.93)	3630.15 (2855.00‒5122.49)	*p* = 0.047
**Total number of Complications**^a^	None	1533.23 (1373.83‒1807.95)	3060.09 (2368.56‒5119.55)	1752.47 (1489.29‒1908.96)	*p*<0.001
	1	7670.51 (4553.87‒11,294.61)	11,589.96 (3855.02‒38,726.75)	8999.25 (4553.87‒11,589.97	*p* = 0.462
	2	26,634.91 (14,702.51‒50,553.79)	19,894.71 (13,887.08‒33,574.03)	22,636.35 (15,168.25‒32,582.37)	*p* = 0,616
**Total number of Procedures**^a^	None	1717.02 (1423.30‒1892.02)	3714.52 (2832.86‒6009.13)	1865.64 (1692.25‒2155.15)	*p* = 0.082
	1	25,396.33 (9066.07‒64,280.24)	11,589.96 (4958.47‒18,112.93)	14,937.61 (9066.07‒23,596.21)	*p* = 0.082
	2 or more	25,976.42 (1399.05‒50,553.79)	33,584.33 (22,485.54‒53,724.14)	33,584.33 (15,592.94‒53,724.13)	*p* = 0.643
**Total**		1793.33 (1489.29‒1976.67)	5446.56 (3815.56‒7421.93)	2113.99 (1847.79‒2609.59)	*p*<0.001

There is significant difference in cost within the category.

a *p* < 0.001.

b *p* = 0.004). For Age group, the reference group was 18‒40 years; For Comorbidities, complications and procedures, the reference group was None Group. ICU, Patients admitted in Intensive Care Unit; MV, Patients submitted to Mechanical Ventilation; 95% CI, 95% Confidence Interval; p-value based on Mann-Whitney U test for independent samples.

**Table 4:** Daily cost according patient acomodation and MV necessity.

Patient Acomodation	Daily Cost in US$
Global Daily Cost	728.46
GMW daily cost	297.53
ICU daily cost	969.72
ICU daily cost without MV	567.40
ICU daily cost with MV	1223.89

GMW, General Medical Ward; ICU, Intensive Care unit; MV, Mechanical Ventilation.

The average cost of patients in the ICU demanding MV was higher than that in any other group. In addition, death was associated with higher costs compared with patients discharged alive (*p* < 0.001). Mean (95% CI) cost for patients discharged alive and patients deceased was US$ 5475.53 (3692.91–7258.14) and US$ 12,955.19 (8106.61–17,803.76), respectively.

Daily hospital costs, which include the costs of ICU and/or general medical ward bed services and nursing services, represented 49% of all presented costs. The medical costs, which included all physician visits and procedures represented only 9.7%. Medications/materials (all medications, therapeutic diets, personal protective equipment, and hemodialysis materials) and oxygen costs represented 26.5% and 13.6%, respectively. Laboratory examinations, imaging, and blood products accounted for 1.2%.

**Fig. 2:**
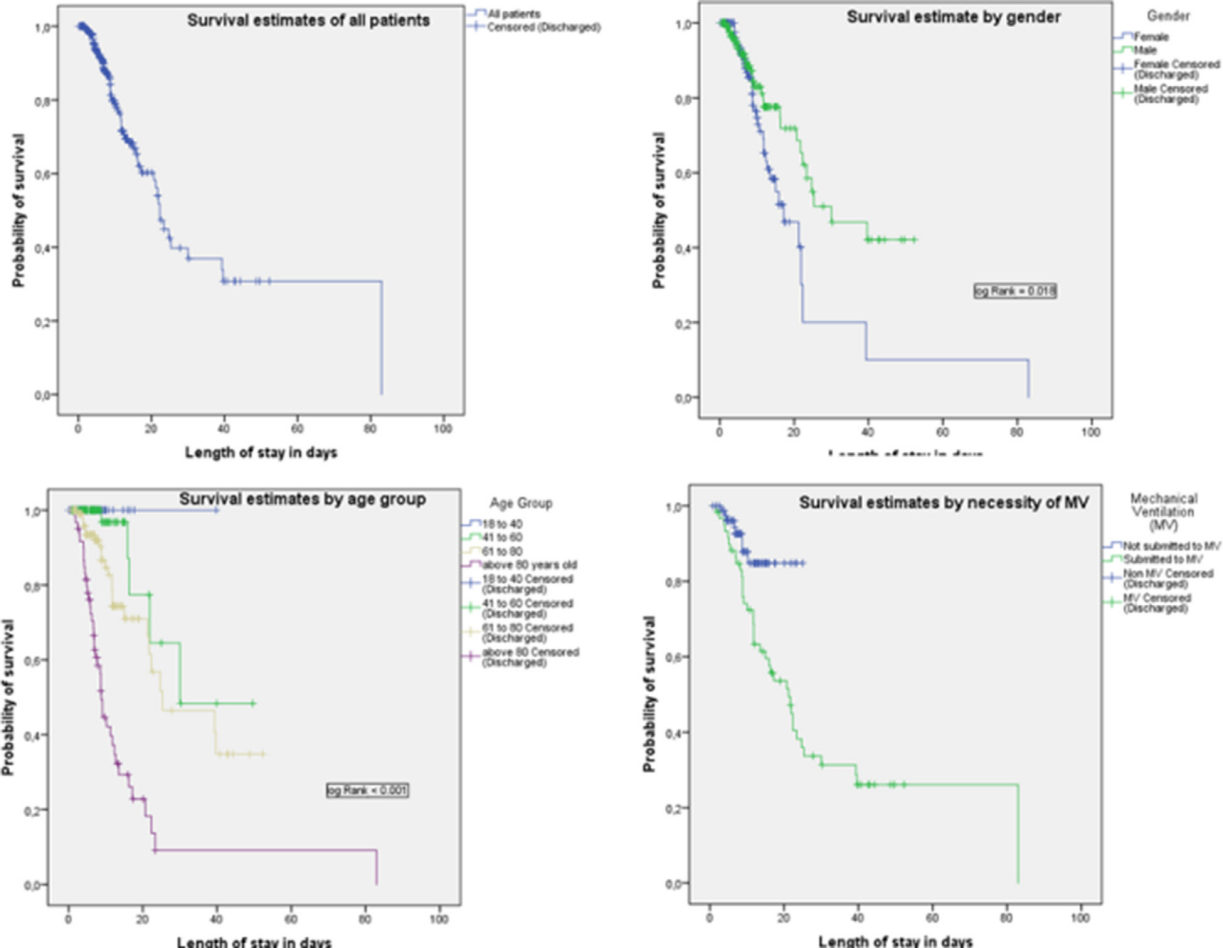
Survival curve analysis.

The global survival analysis and survival curves according to sex, age group, and MV need are presented in Fig. 2. There was a higher probability of survival for male patients (log-rank = 0.018) with a median difference of 13 days, but the difference in the survival probability became evident only after 10 days of LOS. The differences in survival probability for each age group were noticeable from the beginning of the survival curve plot. Although there may seem to be no difference when considering only groups aged 40 to 60 years and 61 to 80 years, survival analysis points to a significant difference between these two groups (Log Rank = 0.038). MV was a risk factor associated with a lower survival rate, particularly after 10 days of MV.

Survival analysis according to total expenditure is presented in Supplement File 1.

## Discussion

The number of patients with COVID-10 which require hospitalization ranges from 5% to 20%, and at least 20% of hospitalized patients require ICU admission.^24^^,^^25^ Age and comorbidities are important predictors of LOS and outcomes.^26^^,^^27^

The healthcare system in Brazil is composed of both private (paid by health insurance or out-of-pocket) and public providers, which are part of the National Health System (SUS) and paid by the Brazilian government. While the public health system is available to all Brazilian citizens, public providers are accessible to 24.4% of the Brazilian population.^28^ Between February and December 2020, the SUS registered 462,149 hospitalizations due to COVID-19 with a total expenditure of US$ 433,979,144.48. Importantly, SUS spent 85% of this amount on hospital services, corresponding to 15% of the expenditures with all hospitalizations in the country.^29^ Although private providers are accessible only to 24.4% of the Brazilian population, it finances almost 50% of all the money spent with direct medical costs in the country.^28^

Economic data from the private health sector in Brazil have not been published so far, and very few studies have analyzed data from the public health sector. One study performed a partial economic evaluation (cost of illness), with suspected, probable, or confirmed consecutive COVID-19 from March 30th to June 30, 2020, analyzing data from hospital admission until final clinical outcome using data from Hospital das Clínicas of the University of Sao Paulo (HCFMUSP), the largest public hospital complex in Latin America.^26^ 3254 Patients with suspected or confirmed COVID-19 were included, and a total of 1683 (51.7%) patients were admitted to the ICU. At the end of the study, there had been 2016 (62%) discharges, 939 (28.9%) deaths, 278 (8.5%) transfers to other facilities, and 21 (0.6%) patients remained hospitalized.^26^ The results of this study presented an average cost per admission of US$ 12,637.42 (US$ 20,002.80 for admissions that included ICU stays at any point and US$ 4839.57 for those who did not), and the overall daily cost was US$ 919.24.^26^ Although it is hard to make direct comparisons mainly due to the use of different perspectives and no standard cluster for comparisons between patients (such as DRG – Diagnosis Related Group), our study suggests that private providers seem to have a lower average cost per admission (US$ 12,637.42 vs. US$ 7060,00), and a lower mortality rate (28.9% vs. 20.5%). The number of comorbidities and disease severity may have influenced the final outcomes, as the HCFMUSP is the reference hospital for severe cases. On the other hand, MV patients seem to be less expensive for the public provider (US$ 1063.22 vs. US$ 1223.89). The costs were recorded in Brazilian reais (R$) in both studies and converted into US dollars (US$). From March 30th to June 30th, US$ 1 was worth an average of R$ 5.55 and from March to December, US$ 1 was worth an average of R$ 5.33. Both health providers presented higher costs for the elderly, and non-medical costs represented more than 80% of the cost for the public and more than 90% for the private provider. One of the most impressive differences observed between the two studies was the fact that for the public provider, death was an outcome associated with lower costs, probably due to shorter LOS, while our findings suggest a higher cost for final outcome death when compared to final outcome discharge.^26^

The National Commission of Incorporation of Technologies for SUS (Conitec) evaluates the cost-effectiveness of new technologies in the SUS portfolio. has presented simulations of hospitalization costs of COVID-19 patients.^30^ GMW cases are estimated to cost US$ 1227.55 per case and ICU cases were estimated to cost US$ 9935.77 per case.

It is even more difficult to compare COVID-19 costs across countries. China has presented a cost of US$ 6827.00 per case in general in a small series of 70 observed cases.^31^ Saudi Arabia reported a mean (SD) cost per patient for those admitted to the GMW and ICU of US$ 11,387.86 (7949.66) and 21,178.21 (14,839.38), respectively.^8^ An estimation showed a total direct medical cost in the USA to be project to range from US$ 163,4 billion to US$ 654,0 billion. The median direct medical cost of caring for a single hospitalized patient was estimated to be US$ 14,366.00.^5^ In Sweden, it is expected that the total direct medical cost bill will reach US$ 2 billion.^32^ The economic burden of COVID-19 in Iran reported direct medical costs of US$ 1.8 million just between March and July, 2020.^6^ In Turkey, the cost of hospitalization and intensive care unit stay were calculated as US$ 19,133.98, and US$ 47,872.08, respectively.^7^

The burden of disease is certainly not lower in not-so-wealthy countries. Over the period of March to November 2020, 2543 COVID-19 cases treated in Ethiopia showed a mean cost per patient to be US$ 1473.00 (95% CI 1197.00–1750), but the cost varied according to disease severity: the mean cost for moderate, severe and critical cases were US$ 1266.00 (998.00–1534.00), US$ 1545.00 (1413.00–1677.00) and US$ 2637.00 (1788.00–3486.00), respectively.^33^ Economic evaluations are usually impacted by local socioeconomic conditions, mainly related to resource availability, currency rates, access to modern technology and medications, as well as different cost of medical staff and all health professionals. However, many challenges posed by the current pandemic are global, such as the worldwide shortage of Personal Protective Equipment (PPE) and mechanical ventilators, which have struck Brazil and everywhere very hard.^26^^,^^34^

Another way to analyze the costs of COVID-19 hospitalization is to compare such costs to those of other infectious diseases. Hospitalization of COVID-19 cases is, on average, over four times more expensive than the hospitalization of symptomatic influenza cases and approximately 5.5 times more expensive than a pertussis case.^5^^,^^35^^,^^36^ In Ethiopia, the cost of COVID-19 hospitalization is estimated to be much higher than that reported for hospitalized patients with measles (US$ 150.00 per severe case). AIDS patients in Ethiopia, considering all costs with inpatients and ambulatorial cases expend only US$ 213.00 for annual treatment of a case.^33^ Saudi Arabia presented mean direct medical costs per patient not significantly different from the one reported for the management of a MERS-CoV patient in Saudi Arabia (around US$ 13,000.00).^8^^,^^37^

Our study has some limitations because it took place in a single city, which could limit its generalizability, and it was performed from a single private health provider perspective. Indirect costs and intangible costs associated with losses in productivity and mortality were not included in our analysis. Estimations of the impact in quality of life and utilities of different COVID-19 scenarios have not yet been published for the Brazilian population. Sensitivity analysis was not performed. However, our data were analyzed using standardized and reproducible methods that make the results applicable to other private health providers. The use of National Directives for Economic Studies and the Use of Diagnosis Related Groups ensured that the cost analysis of these admissions was precise, homogeneous, and less subject to errors. In 2020, there was no influence of the vaccine or new variants of the virus. No specific treatment was available.

## Conclusions

The hospital costs of COVID-19 patients are a major part of the economic impact of the pandemic on healthcare. Patients admitted with COVID-19 in the private hospitals analyzed in the paper point to great economic impact, mainly in the elderly and high-risk patients. It is key to better understand such costs in order to be prepared and to make wise economical decisions during the current global health emergency.^38^ A significant number of patients with COVID-19 require hospitalization and many potential treatments will focus in the management of such patients, which further increases the relevance of an economic evaluation from the hospital's perspective.

## Authors’ contributions

Concept and design: Jaime Rocha.

Acquisition of data: Jaime Rocha and Irina Riediger.

Analysis and interpretation of data: Felipe Tuon.

Drafting of the manuscript: Juliano Gasparetto.

Critical revision of the paper for important intellectual content: Felipe Tuon and Jaime Rocha.

Obtaining funding: None.

## Financial support

No financial support was provided relevant to this article. Felipe Tuon is a CNPQ Reseacher.

## Data availability statement

Deidentified participant data and a data dictionary defining each field in the set will be made available upon request. They will be sent by the corresponding author after approval of a proposal with a signed data access agreement. The data are available for publication.

## Conflicts of interest

The authors declare that they have no affiliations with or involvement in any organization or entity with any financial interest in the subject matter or materials discussed in this manuscript. There was no financial support.

## Appendix Supplementary materials

Image, application 1

Image, application 2
