# Supplementary figures and images for: COVID-19 in real world: Survival and medical costs of hospitalized patients in Brazil´s first wave
Source: Braz J Infect Dis. 2023 May 15;27(4):102778. doi: 10.1016/j.bjid.2023.102778 (PMC10183623; doi:10.1016/j.bjid.2023.102778)

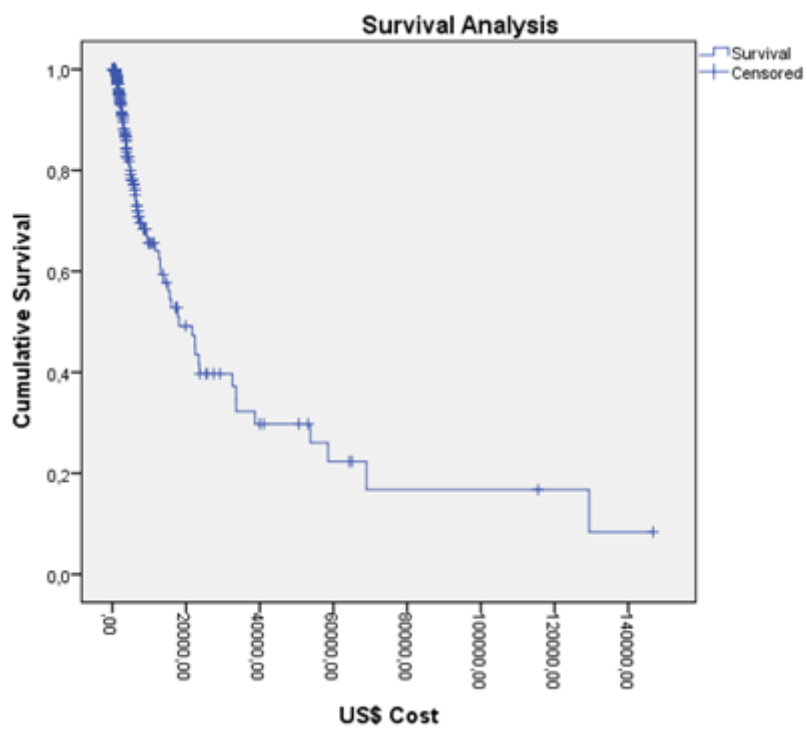

Supplement: Supplementary file 2 [file mmc2.pdf]
